# Survival of Influenza A(H1N1) on Materials Found in Households: Implications for Infection Control

**DOI:** 10.1371/journal.pone.0027932

**Published:** 2011-11-22

**Authors:** Jane S. Greatorex, Paul Digard, Martin D. Curran, Robert Moynihan, Harrison Wensley, Tim Wreghitt, Harsha Varsani, Fayna Garcia, Joanne Enstone, Jonathan S. Nguyen-Van-Tam

**Affiliations:** 1 Health Protection Agency, Addenbrookes Hospital, Cambridge, United Kingdom; 2 Division of Virology, Department of Pathology, University of Cambridge, Cambridge, United Kingdom; 3 Division of Epidemiology and Public Health, University of Nottingham, Nottingham, United Kingdom; 4 Health Protection Agency, East Midlands, Nottingham, United Kingdom; University of Hong Kong, Hong Kong

## Abstract

**Background:**

The majority of influenza transmission occurs in homes, schools and workplaces, where many frequently touched communal items are situated. However the importance of transmission via fomites is unclear since few data exist on the survival of virus on commonly touched surfaces. We therefore measured the viability over time of two H1N1 influenza strains applied to a variety of materials commonly found in households and workplaces.

**Methodology and Principal Findings:**

Influenza A/PuertoRico/8/34 (PR8) or A/Cambridge/AHO4/2009 (pandemic H1N1) viruses were inoculated onto a wide range of surfaces used in home and work environments, then sampled at set times following incubation at stabilised temperature and humidity. Virus genome was measured by RT-PCR; plaque assay (for PR8) or fluorescent focus formation (for pandemic H1N1) was used to assess the survival of viable virus.

**Conclusions/Significance:**

The genome of either virus could be detected on most surfaces 24 h after application with relatively little drop in copy number, with the exception of unsealed wood surfaces. In contrast, virus viability dropped much more rapidly. Live virus was recovered from most surfaces tested four hours after application and from some non-porous materials after nine hours, but had fallen below the level of detection from all surfaces at 24 h. We conclude that influenza A transmission via fomites is possible but unlikely to occur for long periods after surface contamination (unless re-inoculation occurs). In situations involving a high probability of influenza transmission, our data suggest a hierarchy of priorities for surface decontamination in the multi-surface environments of home and hospitals.

## Introduction

Influenza transmission is well documented in households and other residential settings [1]–[4]. Yet the underlying mechanisms of transmission remain poorly understood and hotly debated [5], [6]. Although transmission by aerosols (particles typically <5 µm in diameter), larger droplets and contact transmission (direct and via fomites) probably all play some role, the relative importance of each is uncertain, which has led to difficulties regarding the provision of evidence-based infection control advice for both pandemic and seasonal influenza [7]. If virus can survive for meaningful periods on surfaces and objects, or alternatively, if surfaces are frequently re-inoculated (e.g. by toddlers), then it is feasible that transmission via fomites might occur.

The potential for transmission of influenza by indirect contact (i.e. via fomites) is linked to the ability of virus to survive in transmissible titres on commonly touched surfaces; however few data exist on this subject. Parker et al (1944) demonstrated improved survival of influenza viruses in the presence of human mucus [8]; and in 1962, Buckland demonstrated experimentally that influenza virus was inactivated relatively quickly on glass, probably through desiccation [9]. In 1982, widely cited work by Bean et al showed that both influenza A and B, directly applied to stainless steel surfaces or hard plastic, could survive for 24–48 hours, and be transferred, from there to hands, for 24 hours; survival was much shorter on porous materials such as paper and cotton (8–12 hours), with transferability to hands for only 15 minutes [10]. In contrast, Thomas et al, recently demonstrated survival of human seasonal A (H1N1) and A (H3N2) on Swiss banknotes for up to three days, increasing to up to eight days when applied with nasopharyngeal secretions from children (17 days if applied at very high concentration). Although viable virus was recovered at each of these time points, it was noted that virus load declined sharply after the first few days; no other materials were tested [11]. Other studies have detected influenza virus on fomites in homes and health and childcare facilities, using RT-PCR to establish the presence of the viral genome [12]–[14]. However, data obtained using this technique (even quantitatively) do not distinguish adequately between viable and non-viable virus and are therefore problematic to interpret in the context of practical infection control guidance. In another recent study, virus was detected by PCR on commonly touched household surfaces, but only one sample proved culture positive [15]. However, the time from deposition to recovery was not known, nor the extent of any cleaning undertaken.

We evaluate the survival of influenza A (H1N1) viruses deliberately applied to a range of commonly touched household and workplace surfaces, using RT-PCR for genome detection and culture methods to determine viability. We conclude that RT-PCR is only useful to demonstrate the absence of virus and that on most surfaces, virus viability drops rapidly. Nevertheless, on certain non-porous surfaces, viable virus persists for several hours, rendering fomite transmission possible without re-inoculation.

## Materials and Methods

To test the surface survival of influenza virus, we used a variety of materials commonly encountered in the home and workplace, including a hospital setting (Table 1); choice of surfaces to be tested was discussed with the Department of Health, England to ensure relevance to public health policy. These included fibrous materials such as the ubiquitous J-cloth® (Associated Brands) widely used for cleaning, a silver impregnated fabric with known bacteriostatic properties (Toray Textiles Europe Ltd.) of the type sometimes encountered in hospital staff clothing to combat nosocomial bacterial infections, as well as fabric from a child's soft toy. The latter fabric was made of non-absorbent polyester and, although a porous item overall, individual fibres might perform as a non-porous surface. A variety of non-porous plastic surfaces representing objects highly likely to be touched by multiple individuals such as light switch, telephone and keyboard plastics were also tested, as well as porous and non-porous ‘background’ materials such as various wood surfaces, glass, Perspex/plexiglass (poly (methyl methacrylate) - a thermoplastic often used as a light or shatter-resistant alternative to glass) and metals. As a control surface, we used standard laboratory polystyrene culture dishes. As viruses, we used two human H1N1 strains: the laboratory adapted A/Puerto Rico/8/34 (PR8) strain because of ready availability and robust, convenient assay systems with a wide dynamic range, and an isolate of the current 2009 pandemic virus A/Cambridge/AH04/2009 (AH04), as a low passage history representative of a virus likely to be encountered in the current environment. The source and disinfection method used to clean the various surfaces before testing are listed in Table 1.

**Table 1:** Materials tested, source and preparation prior to use.

Material	Manufacturer/source	Classification	Preparation
J-Cloth®	Associated Brands LP, Toronto, Canada	Porous	Autoclave^1^
Silver containing fabric	Toray Textiles Europe Ltd, UK	Porous	Autoclave
Soft toy	Keel Toys Ltd, UK	Porous	Autoclave
Control: Polystyrene 6-well tissue culture dish	Falcon (Beckton Dickinson & Co., UK)	Non-porous	Sterile^2^
Perspex (poly (methyl methacrylate) -transparent thermoplastic	University of Cambridge, Clinical School workshop	Non-porous	Sterile
Window glass	Go Glass, Cambridge, UK	Non-porous	Autoclave
Light switch material	University of Cambridge, Clinical School workshop	Non-porous	Fumigation^3^
Telephone handset (plastic)	University of Cambridge, Clinical School workshop	Non-porous	Fumigation
Kitchen work surface	Brushwood Ltd, Great Shelford, UK	Non-porous	Fumigation
Computer keyboard	University of Cambridge, Clinical School workshop	Non-porous	Fumigation
Pine (unsealed)	Local work surface manufacturer, Cambridge, UK	Porous	Fumigation
Varnished oak	Local work surface manufacturer, Cambridge, UK	Non-porous	Fumigation
Unvarnished oak	Local work surface manufacturer, Cambridge, UK	Porous	Fumigation
Stainless steel	University of Cambridge, Clinical School workshop	Non-porous	Fumigation
Aluminium	University of Cambridge, Clinical School workshop	Non-porous	Fumigation

1. Autoclaved 121°C, 15 minutes.

2. Received sterile in packaging from manufacturer.

3. Fumigation in a CLIII room using a Laycock Fumigator (Tolbest Ltd).

### Viruses and other materials

Human influenza A virus PR8 (Cambridge lineage) was grown in embryonated hens' eggs and harvested at a titre of 9×10^8^ pfu/ml. For inoculation of the surfaces, the virus was diluted 1∶10 in 1% BSA and serum free media (Dulbecco Modified Eagles Medium, DMEM, Gibco, UK). This represented a viral titre approximating 1.5×10^8^ TCID50/ml, just above the upper end of titres reported for human shedding [10], [11]. Preliminary experiments established that virus survival was improved by the addition of extra protein to the suspension. We tested 0.5% or 1% BSA as well as four preparations of artificial mucus produced from pig stomach mucosa (NBS Biologicals), pig stomach mucin types II or III or bovine sub maxillary glands mucin, type I-S (all from Sigma Aldrich). 1% BSA had the largest effect on titre and duration of survival, followed by the bovine mucin (data not shown). In the interests of simplicity and reproducibility, 1% BSA was therefore used in all subsequent experiments.

To test a 2009 pandemic influenza A (H1N1) virus strain on selected surfaces, a clinical isolate designated influenza A/Cambridge/AHO4/2009 (AH04) was passaged once in MDCK cells and then grown in Caco-2 cells (colorectal adenocarcinoma cells, ATCC HTB-37™). The virus is a recent isolate from an immunocompetent patient who was hospitalised briefly at the start of their illness, but recovered. This virus does not form discrete plaques in MDCK cells and could therefore not be titred by this method. Instead, virus stocks were quantified by qPCR for segment 7 [12]. Although this method scores viable and non-viable virus particles alike, preparations of wild type influenza A viruses generally have similar particle∶PFU ratios and quantitative comparison of RT-PCR and other titration methods have shown good agreement [13], [14]. The AH04 stock contained 6.5×10^8^ genome copies/ml and was used at a 1∶10 dilution as for PR8. For comparison, the PR8 stock had a genome titre of 1.6×10^11^ genome copies/ml.

Mouse monoclonal AA5H (Abcam) was used to detect influenza NP by immunofluorescence.

### Survival assay

Surfaces were cut into 2 cm^2^ pieces and sterilised by a variety of means depending on the surface to be tested (e.g. autoclaving, fumigation etc). Sterile surfaces were glued into sterile 6-well tissue culture dishes using cyanoacrylate adhesive (Henkel, UK). Preliminary experiments (data not shown) demonstrated that dried adhesive alone was non-inhibitory to influenza virus. Under the same conditions of temperature and humidity (ranges 17–21°C and 23–24% respectively), 10 µl volumes of virus were applied to six samples of each surface at the same time. Sampling was conducted immediately – time zero – to demonstrate recoverability. A cotton swab was moistened by dipping in 3 ml of virus transport medium (VTM, Remel, UK) and then wiped carefully in 6 different directions for 1 minute across the top of the surface. Keeping everything on ice, the swab was placed into the tube containing the residual (3 ml) volume of VTM and vortexed for 1 minute. After this, the sample was split directly into 6 eppendorf tubes and stored on dry ice prior to freezing at −70°C. The remaining samples in the plate were kept in a plastic, lidded box at constant temperature and humidity. At 4, 9, 24, 48 and 72 hrs, further samples were taken and stored. After initial experiments it was clear that the virus did not survive in detectable amounts for more than 24 hrs, therefore for the majority of the experiments only the first 4 time points (0, 4, 9, and 24 hrs) were taken. Initial experiments with PR8 virus also showed that loss of virus on the swab was not a major factor, with recovery of virus at time zero from polypropylene surfaces approaching 50% of initial titre (data not shown).

### Virus titration

The qRT-PCR assay used has been described previously [12]. In brief, primers and probes to the Matrix gene of influenza A were used to detect the presence of the virus on the surfaces. Samples from all time points were stored and then extracted. Virus genome was amplified to check that the quantity of virus deposited on the different surfaces was consistent and to determine whether any of the surfaces affected the genome over time.

Plaque assays were performed as previously described in MDCK cells using Avicell overlays [15], [16], in duplicate or where possible in triplicate.

To detect AH04 virus by fluorescent focus assay, infectious material from swabs was first allowed to amplify by inoculation into 1×10^6^ MDCK cells and incubation for 48 h. Supernatant virus was then diluted 1∶10 in serum free DMEM and 250 µl used to inoculate 1.5×10^5^ MDCK cells in a 24 well tissue culture plate. After virus absorption, the cells were overlaid with 1 ml serum free DMEM media containing 1 µg/ml Worthington's trypsin and 0.14% BSA and incubated overnight at 37°C. The following day they were fixed with 4% formaldehyde in PBS, permeabilised by the addition of 0.2% Triton ×100 in PBS for 5 minutes at RT and fluorescently stained with anti-NP monoclonal antibody and counterstained for DNA with 4,6-diamino-2-phenylindole (DAPI) as previously described [17]. Cells were examined blind by two people and scored semi quantitatively for the presence of infected cells using a standardised schema (− no fluorescence seen; +/− some fluorescence seen (<5% cells infected); +5–10% cells infected; ++ >10% cells infected). The literature indicates immunofluorescence to be at least as sensitive in general as plaque assay [13], [18], [19]. Confirming this, tests using serial dilutions of known quantities of PR8 virus, our method reliably detected 20 PFU of virus in the original sample prior to amplification and 50% of the time detected 2 PFU (data not shown).

## Results

**Figure 1:**
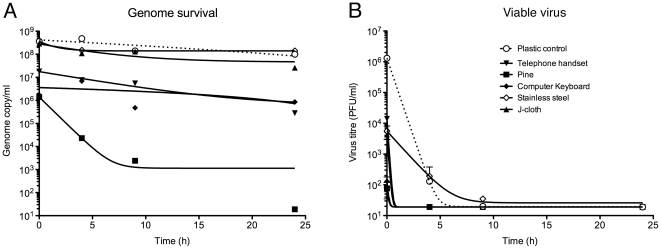
Surface survival of PR8 virus. Virus inoculated onto various surfaces was titred by (A) for genome by RT-PCR or (B) viable virus by plaque assay at the indicated times. Data points were fitted with models for one-phase exponential decay using the program Prism 5 (Graph Pad Software). Lower limit of detection values were used in place of zeros. Values in (A) are from a single determination, while (B) is the mean ± SEM of two or three replicates. Dashed lines indicate the limit of detection.

To test the surface survival of the virus genome, replicate samples of the various materials were inoculated with 10 µl samples containing 1×10^6^ PFU of virus and incubated for defined periods of time before sample recovery was attempted by swabbing. It was noted that the liquid was absorbed by the wooden surfaces within 5 minutes whereas a droplet could be seen on non-porous surfaces for considerably longer, although in all cases, surfaces had dried by 7 hours. Material eluted from the swabs was then titred for virus genome by quantitative RT-PCR. For both PR8 (Fig. 1A, Table 2) and AH04 (Table 3) viruses the results were unambiguous. On most surfaces, the viral genome persisted well, with only around a 10–100 fold drop from the initially recoverable titre after 24 h. The exceptions were unsealed wood surfaces, where both viruses lost genome titre rapidly and on pine surfaces in particular, became undetectable after a few hours. Thus in general, viral RNA survives well for at least 24 h and few surfaces had any significant ‘contact effect’ in immediately reducing genome titre.

**Table 2:** Detection of influenza PR8 virus genome on different surfaces.

		Log_10_ reduction in genome copy number after indicated contact time (hrs)			
Surface	Type*	0	4	9	24
J-cloth	P, NM	−0.26	0.16	0.09	0.79
Silver containing cloth	P, M	1.51	1.38	2.30	1.74
Soft toy	P, NM	−0.26	0.23	0.48	0.71
Window glass	NP, NM	−0.18	0.06	0.48	0.38
Light switch material	NP, NM	1.28	1.16	2.86	1.58
Telephone handset	NP, NP	0.95	1.32	1.46	2.76
Kitchen work surface	NP, NM	2.09	2.25	2.00	2.20
Computer keyboard	NP, NM	1.97	1.29	2.44	2.20
Stainless steel	NP, M	−0.35	0.03	0.06	0.06
Aluminium	NP, NM	1.40	1.67	2.12	2.12
Pine	P, NM	2.06	3.84	4.82	>6.9
Varnished oak	NP, NM	1.27	1.16	1.90	2.49
Unvarnished oak	P, NM	1.27	1.48	2.95	3.16
*Plastic control* †	*NP, NM*	−*0.35*	−*0.48*	*0.06*	*0.20*

*P = porous; NP = non-porous; M = metallic; NM = non-metallic.

Values plotted are the log_10_ reduction in genome copy number with respect to the input inoculum. Under the assay conditions, 100% recovery would have resulted in a titre of 1.6×10^8^ genome copies/ml and the lower limit of detection was <20 copies/ml.

† Plastic control = virus applied directly to the tissue culture dish surface.

**Table 3:** Detection of influenza AH04 virus genome on different surfaces.

		Log_10_ reduction in genome copy number after indicated contact time (hrs)			
Surface	Type*	0	4	9	24
Soft toy	P, NM	0.31	0.67	0.70	1.64
Glass	NP, NM	−0.12	0.43	0.38	0.81
Kitchen work surface	NP, NM	0.00	−0.92	0.81	1.37
Computerkeyboard	NP, NM	−0.01	0.70	1.31	1.17
Stainless steel	NP, M	0.24	0.41	0.53	1.38
Pine (unsealed)	P, NM	−0.33	1.45	2.43	>4.5
*Plastic control* §	*NP, NM*	*0.27*	*0.87*	*1.06*	*1.11*

*P = porous; NP = non-porous; M = metallic; NM = non-metallic.

Values plotted are the log_10_ reduction in genome copy number with respect to the input inoculum. Under the assay conditions, 100% recovery would have resulted in a titre of 6.5×10^5^ genome copies/ml and the lower limit of detection was <20 copies/ml.

§ Plastic control = virus applied directly to the tissue culture dish surface.

When PR8 surface viability was assessed by plaque assay, virus inoculated onto a control surface of a tissue culture dish could be recovered efficiently at t0, but thereafter infectivity fell away rapidly with no live virus recovered at 24 h (Table 4). Fitting the data to a one-phase exponential decay model (Fig. 1B) estimated the t_1/2_ of the virus under these conditions to be around 1.5 h. A similar pattern of rapid loss of infectivity was seen when the household surface samples were tested, with the difference that greater initial losses of infectivity ranging between 20-fold (telephone handset) to nearly 4000-fold (unsealed pine) were seen (Table 4). Nevertheless, viable virus was recovered at 4 h (but not later) from the silver-impregnated cloth, soft toy fabric and in trace quantities, from light switch material. The only material (other than the control tissue culture dish) for which even low amounts of viable virus could be detected at 9 h was stainless steel. Thus despite the persistence of the viral genome on a wide variety of household surfaces, PR8 infectivity decayed sharply, with evidence of significant contact effects from some materials; most notably unsealed pine, but also a wide variety of other porous and non-porous surfaces.

**Table 4:** Survival of influenza PR8 on porous, non-porous, metallic and wood surfaces.

		Log_10_ reduction in infectivity after indicated contact time (hrs)			
Surface	Type*	0	4	9	24
J-cloth	P, NM	1.8	>4.2	>4.2	>4.2
Silver containing cloth	P, M	2.9	3.2	>4.2	>4.2
Soft toy	P, NM	1.9	2.8	>4.2	>4.2
Window glass	NP, NM	1.6	>4.2	>4.2	>4.2
Light switch material	NP, NM	2.8	4.2	>4.2	>4.2
Telephone handset	NP, NP	1.3	>4.2	>4.2	>4.2
Kitchen work surface	NP, NM	3.1	>4.2	>4.2	>4.2
Computer keyboard	NP, NM	3.4	>4.2	>4.2	>4.2
Stainless steel	NP, M	1.7	3.2	3.9	>4.2
Aluminium	NP, NM	2.8	>4.2	>4.2	>4.2
Pine	P, NM	3.6	>4.2	>4.2	>4.2
Varnished oak	NP, NM	2.0	>4.2	>4.2	>4.2
Unvarnished oak	P, NM	2.8	>4.2	>4.2	>4.2
*Plastic control* †	*NP, NM*	*0.6*	*3.4*	*4.2*	*>4.2*

*P = porous; NP = non-porous; M = metallic; NM = non-metallic.

Values plotted are the log10 reduction in plaque titre with respect to the input inoculum and are the mean of 2–3 replicates. Under the assay conditions, 100% recovery would have resulted in a titre of 3×10^5^ PFU/ml and the lower limit of detection was 20 PFU/ml.

† Plastic control = virus applied directly to the tissue culture dish surface.

To test whether these findings could be extrapolated to a currently circulating virus, we next tested the survival of AH04 virus, a 2009 pandemic isolate, on a subset of the materials. Unlike PR8, as a recent clinical isolate this virus does not grow to high titres in the laboratory and nor was a workable plaque assay available. We therefore used a fluorescent focus assay in which live virus is detected by immunofluorescent detection of the viral nucleoprotein in infected cells. To boost the sensitivity with which viable virus could be detected, infectious virus present in the swabs was first amplified by growth in MDCK cells before subsequent assay. The assay therefore provides a highly sensitive but semi quantitative measure of virus infectivity, ideally suited to working with low titre samples [13], [19]. By this measure, the AH04 virus persisted for at least 24 h on the control tissue culture dish material, although titres were evidently lower at 9 and 24 h (Table 5). Consistent with the results obtained with PR8 virus, all household surfaces tested showed lower persistence of infectious virus, with none providing recoverable titre at 24 h and the majority failing to produce live material at 9 h. Once again the pine surface showed very rapid inactivation of viability, with no infectivity recovered at 4 h. Thus both an historic virus isolate and an example of the recent pandemic strain fail to survive in high titres for long periods of time on a variety of household surfaces, but with significant survival over shorter time spans on certain materials.

**Table 5:** Survival of influenza AH04 on a representative range of household surfaces.

		Recovery of AH04 virus after contact time (hrs)			
Surface	Type*	0	4	9	24
Soft toy	P, NM	**+^χ^**	**_**	**_**	**_**
Pine (unsealed)	P, NM	**+**	**_**	**_**	**_**
Kitchen work surface	NP, NM	**+**	**+**	**+/−**	**_**
Glass	NP, NM	**+**	**+**	**+**	**_**
Stainless steel	NP, M	**++**	**+**	**_**	**_**
*Plastic control* †	*NP, NM*	***+***	***+***	***+/−***	***+/−***

*P = porous; NP = non-porous; M = metallic; NM = non-metallic.

− no fluorescence seen; +/− some fluorescence seen (<5% cells infected); + 5–10% cells infected; ++ >10% cells infected.

† Plastic control = virus applied directly to the tissue culture dish surface.

## Discussion

Prior to the influenza A(H1N1) pandemic of 2009–10, few data were available with regard to virus survival on different household surfaces. With a few notable exceptions [10], [11], the majority of studies had been carried out based on RT-PCR to detect the presence of the genome [20]–[22]; these shed no light on the presence or absence of viable virus. In this study we sought to provide contemporary data about virus survival on a wider range of materials found in or on household surfaces than previously described in the literature; these exemplars were chosen after discussion with UK pandemic policy makers. However, one limitation is that our study was confined to H1N1 influenza A viruses (PR8 and the 2009 pandemic virus) due to resource issues. However, we know of no evidence to suggest there are substantial differences in survival between human influenza viruses. Moreover, when we compared the survival of PR8 virus with two seasonal isolates of influenza A (A/Solomon Islands/12/5/08 (H1N1) and influenza A/Brisbane/12/5/08 (H3N2), obtained from Professor Alan Hay of the National Institute of Medical Research, Mill Hill), we saw no significant differences, with all three viruses losing plaque titre on a plastic surface with a t1/2 of around 90 minutes (data not shown). We therefore think it is reasonable to generalise from the findings here to other human strains of influenza A. Further studies, especially of influenza B are warranted however.

We applied concentrations of virus (∼1×10^6^ TCID_50_), which were within the range of those reported in the respiratory secretions of naturally infected individuals [5], [10]. In addition, we suspended virus in 1% bovine serum albumin (BSA), reflecting our (unpublished) finding that BSA improved virus survival, and similar findings from Thomas et al [11] using mucus obtained from children. Our experiments were conducted within a narrow range of humidity and temperature conditions consistent with normal human indoor living conditions in temperate zones, and all survival assays were performed in duplicate and where possible triplicate. We used plaque assay techniques and immunofluorescence techniques for PR8 and pandemic viruses, respectively. The differing methodologies used to detect the two strains of H1N1 virus (lower titre inoculum of the pandemic AH04 virus but higher sensitivity detection method) make it difficult to directly compare the survival of the two strains, but we see little to suggest any major difference.

Our data on the survival of the laboratory adapted PR8 virus indicated that viable virus was no longer recoverable in detectable amounts from 9 of 14 (64%) surfaces four hours after deposition; however, contrary to the findings of Bean et al., non-porous surfaces were not consistently more conducive to virus survival than porous ones [10]. Nevertheless, no test surfaces supported detectable virus survival beyond nine hours. Broadly similar outcomes in which infectivity tended to be lost after 4–9 hours were obtained with the recent pandemic isolate AH04. Overall, our results indicate that influenza virus does not remain viable in large quantities on most surfaces in indoor domestic conditions for more than a few hours. Our data are consistent with recent findings from a study of environmental deposition of pandemic H1N1 virus in the homes of infected patients, involving our laboratory, when almost 10% of tested surfaces yielded viable virus [15]. However, in this and similar studies in community settings where environmental samples are taken relatively infrequently and the infectious source remains present, it is not possible to establish the time elapsed since virus deposition [15], [23].

With regard to the testing of specific materials, we examined survival on a range of porous items: a children's soft toy, a silver impregnated fabric with known bacteriostatic properties of the type sometimes encountered in hospital staff clothing to combat nosocomial bacterial infections, and a branded cleaning cloth (J cloth®, Associated Brands). We hypothesised that the inclusion of an antimicrobial agent, Microban® (Microban International Ltd) in the J cloth might inhibit viral growth. Microban® is based on triclosan and has been demonstrated to have anti-bacterial and anti-fungal activity; it has not however, been demonstrated or claimed to be anti-viral. Notwithstanding, in our laboratory setting, some constituent or quality of the J cloth® appeared to limit virus survival to under 4 hours. The result for the silver impregnated fabric also deserves further comment. Whilst silver has been demonstrated to have bacteriostatic properties, it has not been documented to show antiviral activity. Our data would tend to suggest that it is not significantly inhibitory to influenza A.

Surfaces that allowed PR8 virus to survive longest (between four and nine hours) included light switch material (polyvinyl chloride) and a computer keyboard. Interestingly these are likely to be the materials from which the most frequently touched communal household objects are made. Both PR8 and pandemic viruses survived less than four hours on all of the wood surfaces tested. This may have been due to a number of factors including porosity of the surface, oils in the wood or a potentially virucidal ‘contact effect’ of varnish finishes. Pine oil in particular has been demonstrated to have virucidal activity against respiratory viruses [24]. Our findings suggest they are not hospitable environments for enveloped viruses.

As observed in other studies, we found that stainless steel supported the viability of influenza viruses longer than other tested metals. Metals have been demonstrated to have low levels of anti viral activity [25]–[27]; and stainless steel has previously been demonstrated to support influenza virus viability for longer than that of copper [28]. Confirmation of these results raises questions about the use of stainless steel in healthcare and daycare settings in particular.

In conclusion, testing two H1N1 strains of influenza A (one of which was a 2009 pandemic virus) demonstrates that in an environment that is consistent with indoor domestic settings in temperate zones, virus deposited onto the touched environment is likely to survive up to a few hours, though rarely more than nine hours, on the vast majority of surfaces. Metallic and non-metallic non-porous materials pose the greatest risk and should be targeted for frequent cleaning if situated in close proximity to patients infected with influenza virus; fortunately the latter are also more conducive to surface cleaning with a wide variety of simple cleaning agents [12]. Whilst our data suggest that the risk of virus transmission might last several hours after deposition, we generated very little data suggesting that appreciable amounts of virus survived much beyond nine hours. This probably means that frequently touched environments such as classrooms, offices and living rooms, which are then left unoccupied overnight, will not contain much viable virus on surfaces by the next morning. Nevertheless, the data still support frequent cleaning of commonly touched items and surfaces throughout the working day, particularly when symptomatic persons are present, for example in physician waiting rooms. In terms of cleaning regimens, one critically important consideration is that survival of virus in high titres for prolonged periods is not necessary for fomite transmission if surfaces are frequently re-inoculated (e.g. by toddlers). However the contribution of such indirect transmission relative to respiratory droplets directly from one person to another or relative to aerosol transmission remains unknown.
